# The Phonograph in Medicine

**Published:** 1891-10

**Authors:** 


### The Phonograph in Medicine.

The applicability of the phonograph to the record and demonstration of defects in speech has been well illustrated during the past week at the Royal Medical and Chirurgical Society and at the Hunterian Society.

At the first named, Dr. Hale White and Mr. Golding-Bird were enabled by means of this instrument to allow the Fellows present to hear the curiously defective speech of two children, and to contrast this with the improvement effected by treatment for the subjects were present, and after the phonograph had given their past utterances, their present speech was demonstrated *viva voce.*

The papers read by the above gentlemen and that by Dr. F. Taylor led to an instructive debate, which was further illustrated by some marked cases introduced by Dr. Hadden. The outcome seemed to be that these defects in articulation are probably of central origin, and not due to any mechanical interference with the organs of speech. Whether, as suggested by Dr. Langdon Down and Mr. Spencer Watson, the defect was primarily one of audition is a question certainly worthy of consideration. Another point raised was whether the defect should be considered one of speech or language, and some exception was taken by Drs. Taylor, Pye-Smith and others, to the use of the term "idioglossia," which, however, was ably defended by Dr. Hale White. The other phonographic demonstration at the Hunterian Society was by Dr. Hughlings Jackson, who thus reproduced the characteristic speech of a subject of cerebro-spinal sclerosis. There can be little question that the phonograph will ultimately prove very useful, especially in the preservation of certain anomalies of articulation, and its further extension to other sound phenomena in the range of clinical medicine may be justifiably hoped for.—*The Lancet.*

